# The adaptive value associated with expressing and perceiving angry-male and happy-female faces

**DOI:** 10.3389/fpsyg.2015.00851

**Published:** 2015-06-22

**Authors:** Peter Kay Chai Tay

**Affiliations:** School of Social Sciences, Singapore Management University, Singapore, Singapore

**Keywords:** facial emotion, affect, evolution, sexual roles, sexual selection, happiness, anger

## Abstract

Facial expressions are valuable for conveying and understanding the inner thoughts and feelings of the expressor. However, the adaptive value associated with a specific expression on a male face is different from a female face. The present review uses a functional-evolutionary analysis to elucidate the evolutionary advantage in the expression and perception of angry-male and happy-female faces over angry-female and happy-male faces. For the expressors, it is more advantageous for men to show angry facial expression as it signals dominance, averts aggression and deters mate poaching; it is more advantageous for women to display happy facial expression as it signals their willingness for childcare, tending and befriending. For the perceivers, those sensitive to angry men avoid being physically harmed while those sensitive to happy women gain social support. Extant evidence suggests that facial structure and cognitive mechanisms evolved to express and perceive angry-male and happy-female faces more efficiently compared to angry-female and happy-male faces.

Emotional faces represent a source of important social information. Particularly, one’s intentions can be readily communicated by displaying relevant facial expressions and others’ intentions can be revealed by studying their facial expressions. However, not all emotional faces are associated with the same adaptive value and cognitive resources are limited (e.g., Todd et al., 2005). Evolutionary forces may hence direct physiological and cognitive resources toward emotional faces that confer greater adaptive value. As such, the expression and perception of a facial expression displayed on one sex may be more efficient compared to the same facial expression on another sex. In this paper, I elucidate the adaptive value associated with the expression and perception of the angry and happy emotions for male and female faces based on a functional-evolutionary analysis. I argue that angrymale and happy-female faces are expressed and perceived more efficiently compared to angry-female and happy-male faces.

## A Functional-Evolutionary Perspective on Emotional Faces

Using the functional-evolutionary approach to examine findings on facial expressions can shed light on why some emotional faces are expressed and perceived more quickly and accurately. Other than communicating internal thoughts and feelings (Keltner, 1995; Öhman and Mineka, 2001; Tracy and Robins, 2004), facial expressions also signal what behaviors are to follow (Andrew, 1963; Chevalier-Skolnikoff, 1973), leading to evolved facial structures and cognitive mechanisms that are consistent with the adaptive value conferred by these facial expressions. Adaptive value in the current paper refers to the notion that the evolutionary advantage associated with expressing and perceiving a facial expression on a man is different from a woman. For instance, it may be more advantageous to attend to a woman with a happy facial expression compared to a man with a happy face because women generally tend to lend nurturant and caring support (Eagly and Crowley, 1986), and the happy facial expression signals this intention (e.g., social smiling, Baron-Cohen, 2003; Shiota et al., 2004). Relatedly, it has been suggested that women tend to possess better empathizing abilities than men (Manstead, 1992; Baron-Cohen, 2003), suggesting that women also demonstrates the ability to nurture and care. Thus, evolved mechanisms would be geared toward expressing and perceiving happy-female faces more than happy male faces.

Shariff and Tracy (2011) proposed that emotional expressions originally evolved to facilitate actions contingent on the demands of the environment and later co-opted to communicate social information. Evidence suggests that the expression and perception of facial expressions has evolutionary origins. Infants show greater interest for emotional faces over other stimuli (Yamaguchi, 2000); the universality in the expression and perception of facial expressions (Ekman, 1994; Izard, 1994); the automatic perception of facial expressions, and the observation that certain facial expressions such as happiness and anger is shared with primates (Öhman, 2002) suggest that our cognitive mechanisms are shaped by evolution to process emotional faces efficiently.

The evolutionary advantage in attending to negative emotional stimuli has been explained by the negativity bias. Baumeister et al. (2001, p. 325; see also Vaish et al., 2008) mentioned that “it is evolutionarily adaptive for bad to be stronger than good. We believe that throughout our evolutionary history, organisms that were better attuned to bad things would have been more likely to survive threats and, consequently, would have increased probability of passing along their genes.” This tendency to attend to negative valence stimuli is supported by a huge body of research (see Hamann, 2001). With respect to facial expressions, threatening faces such as angry faces captures attention more than emotionally positive or neutral faces (Esteves et al., 1994; Bannerman et al., 2010; Feldmann-Wüstefeld et al., 2011; Huang et al., 2011). Expressing and perceiving threatening faces is important for survival and evidence suggests that cognitive resources are recruited to rapidly show and identify emotional faces that are indicative of threats. For instance, threatening faces are automatically attended to and implicate the amygdala to prepare the individual for the “fight-or-flight” responses (Öhman, 2002). It is noteworthy that most theories explicating the adaptive value for emotional faces focus more on self-protective goals rather than self-enhancing goals. Consequently, the literature is biased toward the investigation of the adaptive value associated with emotionally negative facial expressions, leaving little investigation on emotionally positive facial expressions (e.g., Goos and Silverman, 2002).

Although research findings suggest that emotionally negative faces such as angry faces draw more attention (e.g., Frischen et al., 2008; Huang et al., 2011), recent evidence suggests that positive emotion faces such as happy faces may be detected more readily than angry faces (Becker et al., 2011; Craig et al., 2014). Taken further, the functional-evolutionary perspective suggests that it may be more adaptive to be sensitive to angry men than angry women because angry men possess greater physical threat because of their bigger stature compared to women (Lassek and Gaulin, 2009). In contrast, it may be more adaptive to be sensitive to happy women because happiness signals their intention to befriend and provide care while sensitivity toward happy men may not be as beneficial because they are less likely to provide such social support (Taylor et al., 2000). In the current paper, this phenomenon is termed as the angry-male and happy-female advantage. The following elucidate this phenomenon using a functional-evolutionary analysis.

Intersexual and intrasexual selections are two related evolutionary processes that potentially contribute to the angry-male and happy-female advantage. Intrasexual selection refers to the “tendency of members of one sex to compete with one another for access to members of the opposite sex” and intersexual selection refers to “the tendency of members of one sex to preferentially choose as mates certain members of the opposite sex (Buss and Barnes, 1986, p. 559).” Due to sex differences in terms of greater parental effort for women and greater parental uncertainty for men, women are more intersexually selective and men are more intrasexually competitive (see Stewart-Williams and Thomas, 2013). Particularly, parental effort is greater for women because it involves a lengthy pregnancy followed by a period nursing the young (Clutton-Brock and Vincent, 1991). This results in women being more selective of their mates (i.e., intersexual selection), preferring mates who could offer protection and acquire resources during their pregnancy and childcare period. Although long-term mate attributes such as intelligence and health are equally important for both men and women (Buss, 1989), men are also intersexually selected for their dominance and aggression because these attributes are related to abilities in providing protection and acquiring resources for their mates (Buss, 1989; Ellis, 1992; Ketelaar et al., 2012). This may have led to a tendency for men to express anger which signals dominance and aggression. Relatedly, it was reported that men and women who showed happiness were perceived as less and more sexually attractive respectively (Tracy and Beall, 2011), and the tendency to smile has been suggested to be mediated by testosterone levels (Ellis, 2006; Ellis and Das, 2011).

Intrasexually, men compete with each other for access to potential mates and engage in mate guarding (Buss and Kenrick, 1998; Geary, 2000; Wood and Eagly, 2002), and because parental certainty is not guaranteed for men (Alexander, 1979; Daly et al., 1982), they may engage in aggressive and dominating behaviors that prevent cuckoldry. As such, angry facial expression may serve as cues of threats and aggressions to mate competitors and potential mate poachers. Concurrently, cognitive system that is sensitive in detecting such cues can prevent perceivers from engaging in mate poaching. In line with this notion, research found that men engage in more direct aggression especially those that are violent in nature (Daly and Wilson, 1988; Archer, 2004). Considering the greater tendency for men to inflict violence and the larger stature of men compared to women (Gaulin and Boster, 1985; Lassek and Gaulin, 2009), this could have given rise to cognitive systems that readily detect and attend to men expressing anger in order to avoid physical harm. Conversely, it is not as exigent to detect and attend to women expressing anger because confrontations with aggressive women rarely result in serious injuries or deaths (Campbell, 1999).

Happy facial expression has been found to subserve a number of social functions such as signaling that all is well (Scherer, 1988; Preuschoft and van Hooff, 1997; Hess et al., 2007), concealing negative emotions (Levine and Adelman, 1993; Ansfield, 2007), showing both dominance and affiliation (Knutson, 1996), and may be used to show sexual interest among women (Levine and Adelman, 1993; Tracy and Beall, 2011). Happy facial expression also indicates one’s willingness to connect interpersonally especially among women (Hess et al., 2005; Parkinson et al., 2005). On the basis of evolutionary theories, being sensitive to happy women appears to be more adaptive compared to happy men. Specifically, women are universally more involved in childcare than men (Babchuk et al., 1985; Wood and Eagly, 2002; Stewart-Williams and Thomas, 2013). As such, women who possess characteristics that are suited for these activities are intersexually selected for. In addition, as women offer social emotional support to each other during stressful times (Taylor et al., 2000), women who exhibit interests in the well-being of others may also be intrasexually selected for. Indeed, extant evidence suggests that women are more likely than men to lend social support as well as receiving social support from female close others (Wethington et al., 1987; Ogus et al., 1990; McDonald and Korabik, 1991). This is consistent with the observation that oxytocin, which is more abundant in women compared to men, may contribute to learning and memory pertaining to social relationships (Popik et al., 1992; De Wied, 1997).

## Empirical Evidence for the Angry-Male and Happy-Female Advantage

Several existing theories show converging support for the angry-male and happy-female advantage. It is noteworthy that while sex stereotypic emotions may not necessarily reflect actual emotional experience (Fischer, 1993), sex differences in terms of the expression and perception of happy and angry facial expression have been observed. On one hand, the theory which outline women’s tendency toward “tend-or-befriend” rather than “fight-or-flight” suggests that women commonly show emotions related to their caregiving roles and sex stereotypes such as happiness, fear and sadness (Grossman and Wood, 1993; Plant et al., 2000; Taylor et al., 2000). On the other hand, there are theories suggesting that men tend to show negative emotions, particularly anger, more than women because of the association with their protective role and competitiveness (Grossman and Wood, 1993; Brody and Hall, 2000; Plant et al., 2000; Fischer et al., 2004).

A huge body of empirical evidence support this angry-male and happy-female advantage in terms of perception (Goos and Silverman, 2002; Hess et al., 2004, 2009; Becker et al., 2007; Pixton, 2011), detection (Becker et al., 2007; Aguado et al., 2009; Kenrick et al., 2010; Amado et al., 2011; but see Becker et al., 2011), expression (Kring, 2000; Fischer et al., 2004), and reaction toward (Dimberg and Öhman, 1996) emotional faces. Generally, individuals tend to express, and are more accurate and faster in responding to angry-male and happy-female faces than other facial expressions. Interestingly, when faces are androgynous, angry and happy facial expressions make the faces more male-like and female-like to the perceivers respectively (Becker et al., 2007; Hess et al., 2009). Consistent with the functional-evolutionary account outlined above, this suggests that male faces induces the “fight-or-flight” action tendencies while female faces prompt the “tend-and-befriend” action tendencies.

In the context of social interaction however, detecting and attending to emotional faces are insufficient. It is also necessary to remember the faces expressing the emotions so as to allow one to respond accordingly in the future. To date, research that investigates sex differentiated facial expressions largely involves the expression, perception and detection of emotions rather than memory (e.g., Becker et al., 2007; Hess et al., 2009). Nonetheless, there is some evidence suggesting that negative emotion male faces and positive emotion female faces are retained better in memory compared to positive emotion male faces and negative emotion female faces (Hofmann et al., 2006; Wang, 2013). Studies also found that angry-male faces are vividly represented in the working memory compared to angry-female faces (Jackson et al., 2009, 2014; Becker et al., 2013).

## Implications and Future Directions

The current review demonstrates that theories from the sociological and evolutionary perspectives concur with respect to the advantage in expressing and perceiving the angry-male and happy-female facial expressions. Particularly, men and women more often express the angry and happy facial expressions respectively, and angry-male and happy-female faces are perceived more efficiently than their counterparts. While extant evidence suggests that both expressor and perceiver factors come into play, it remains unclear if this phenomenon arises more from the expressor’s facial architecture or the perceiver’s cognitive mechanism: Is it because male faces are inherently angrier looking and female faces happier looking, or is it because it is easier for the perceiver to detect male faces when anger is expressed and female faces when happiness is expressed?

Evidence suggests that human faces are sexually dimorphic to the extent that male facial architecture is structurally more attuned to angry expression while female faces more alike happy expression (Becker et al., 2007). This notion is supported by studies which found that sex differences in the perception of angry and happy faces disappear when male and female faces are equated for physical cues of dominance such as thick eyebrows and high forehead (Hess et al., 2004, 2009). Nevertheless, research which demonstrates that the processing of facial expression can be biased by factors such as individual experiences (Fabes and Martin, 1991; LaFrance et al., 2003), and power and social status (Henley, 1977; Hall, 1984) implies that perceiver cognition also plays a role. Thus, more investigations are necessary to detangle the effects between the facial structure of the expressor and the cognitive processes of the perceiver. An interaction between expressor facial architecture and perceiver cognition can be hypothesized. Particularly, the perception of angry-male and happy-female should be observed for individuals who are exposed to sex stereotypic information such as occupation and power differences, only when the facial structure is unambiguously male or female. In addition, facial morphing techniques can be used to examine if it alters the perception of happiness and anger in faces given the sex stereotypic information. Specifically, facial morphs approaching greater maleness and femaleness is likely to accentuate the perception of anger and happiness respectively, particularly for individuals exposed to sex stereotypic information or individuals who hold gendered beliefs.

Emotional faces provide invaluable social cues. A face expressing positive emotions such as happiness suggests that the environment is safe while a face expressing negative emotions such as fear and anger suggests that something is wrong, activating the “fight-or-flight” mechanism (Lang et al., 1997). Because facial expressions are indicative of a person’s intentions and inner thoughts (Frijda, 1982, 1986), the perceiver taps on this emotional information to generate appropriate actions. In particular, facial expressions allow one to decide if the expressor should be approached or avoided.

The approach-avoidance tendency describes the adaptive responses to emotional stimuli (Buck, 1984; Frijda, 1988). Responding appropriately in accordance to the evoked emotion can enhance a person’s survival and social reception. Therefore, encountering an angry-male and a happy-female is likely to induce avoid and approach action tendencies respectively. This is in line with the notions that angry faces signal “stay away” and happy faces signal “approach me” (Becker et al., 2007) and that trustworthy faces are perceived as more happy and at the same time, less angry (Todorov, 2008; Oosterhof and Todorov, 2009). While a small number of studies examined the approach and avoidant tendencies in response to the happy and angry facial expressions (Marsh et al., 2005; Winkielman et al., 2005; Stins et al., 2011), it remains unclear if there are sex differences. Furthermore, these studies are biased toward the investigation of angry facial expressions. Considering that emotionally neutral male and female faces tend to appear angrier and happier (Becker et al., 2007), it is likely that neutral male and female faces would induce avoidance and approach action tendencies respectively. Future studies can examine if the tendency to approach and avoid is related to the maleness and femaleness respectively, and if this effect is mediated by the degree of happiness and anger perceived. In addition, studies suggest that men and women were perceived to be more sexually attractive when they expressed the angry and happy facial expressions or behaviors respectively (Sadalla et al., 1987; Tracy and Beall, 2011). Future research can investigate if expressors also display the associating facial expressions in order to attract mates. It can be hypothesized that men and women will have a greater tendency in displaying the angry and happy expression respectively when intersexual mating motivation is activated. In addition, as intrasexual competition with respect to the display of dominance and aggression is more relevant to men than women, it is expected that mating motivation would lead to greater expression of the angry facial expression toward male competitors among men but not women. Cross-cultural data and physiological measures such as electromyography can be used to investigate these predictions.

## Conclusion

The present review focused on sex differences for the expression and perception of happy and angry facial expressions. A wealth of research findings is consistent with the notion that expressing and perceiving angry-male and happy-female faces is more efficient than angry-female and happy-male faces. This sex differentiated phenomenon for emotional faces may generalize to other discrete facial expressions. For instance, sadness and fear are also associated with female faces while pride is associated with male faces (Hess et al., 2000; Plant et al., 2000). Using a functional-evolutionary approach to elucidate the underpinnings for the expression-sex link represents a fruitful endeavor and gives impetus to new research that can potentially generate more insights for the emotion literature.

### Conflict of Interest Statement

The author declares that the research was conducted in the absence of any commercial or financial relationships that could be construed as a potential conflict of interest.
